# Type I-interferon β induces a strong anti-tumour response in bladder cancer cells

**DOI:** 10.1007/s00432-025-06409-1

**Published:** 2026-01-08

**Authors:** Marlena Hesse, Max Iltzsche, Daniel Nahhas, Christian Thomas, Susanne Füssel, Barbara Kind

**Affiliations:** Department of Urology, Medizinische Fakultät Carl Gustav Carus, Technische Universität Dresden, Fetscherstr. 74, 01307 Dresden, Germany

**Keywords:** Bacillus Calmette-Guéérin (BCG), Bladder cancer (BLCA) cells, Interferon (IFN), Non-muscle invasive bladder cancer (NMIBC)

## Abstract

**Purpose:**

For non-muscle invasive bladder cancer (NMIBC), instillation with Bacillus Calmette-Guérin (BCG) is a standard therapy. With a still unclear mechanism, instillation activates the innate immune system, resulting in an immunological effect on the tumour. The aim of this work was to investigate which bladder cancer (BLCA) cells can be activated by interferon (IFN) exposure.

**Methods:**

The BLCA cell lines RT4 and SW780 were stimulated with IFN-α2, -β and -λ1 over 4–72 h. Quantitative PCR (qPCR) was used to determine the expression of IFN receptor subunits (RS) and selected interferon-stimulated genes (ISGs). Luciferase reporter assay was performed to detect the activation of the IFN responsive element (ISRE). Different signal transduction molecules of the JAK/STAT pathway were assessed by Western Blot to prove its activation in BLCA cells. The viability of the stimulated cells was measured by WST-1 assay and the apoptosis induction by caspase-3/7 assay.

**Results:**

The JAK/STAT pathway was activated via the four RS. Upon long-term treatment, type I and type III IFNs significantly induced increased ISG expression and apoptosis induction of RT4 and SW780 cells, emphasising their antiproliferative and immunomodulatory activity. This activation was mediated by ISRE. IFN-β activated the JAK/STAT pathway with the greatest potency, highlighting its superior efficacy in modulating cellular responses in BLCA.

**Conclusion:**

Activation of the innate immune system has the ability to trigger further infiltration of the tumour microenvironment (TME) with immune cells, which positively influence the TME in its type, density and immunofunctional orientation against BLCA.

**Supplementary Information:**

The online version contains supplementary material available at 10.1007/s00432-025-06409-1.

## Introduction

Non-muscle-invasive bladder cancer (NMIBC) represents approximately 75% of all new cases of bladder cancer (BLCA) (Grabe-Heyne et al. 2023). The standard of treatment for high-grade NMIBC is the transurethral endoscopic tumour resection followed by intravesical treatment with Mitomycin C or the attenuated bacterium Bacillus Calmette-Guérin (BCG). While this treatment regimen is effective for many patients, approximately one third of patients become resistant over time (Green et al. 2021). The therapeutic success of BCG relies on its ability to induce a robust, local inflammatory response within the bladder, leading to a T-helper 1 (Th1) polarised immune-mediated clearance of tumour cells. However, the efficacy of BCG is not universal, and its utility is currently compromised by a chronic and severe global shortage, creating an urgent and unmet clinical need for effective and readily available therapeutic alternatives (Lidagoster et al. 2024).

In the search for such alternatives, the field has logically focused on cytokines that can replicate the immune-stimulating effects of BCG. Interferons (IFNs), particularly type I-IFNs, have been a primary focus. This has led to the clinical use of IFN-α as a salvage therapy in combination with BCG (Luo et al. 1999), (Stricker et al. 1996) and, more recently, to the landmark FDA approval of nadofaragene firadenovec (Adstiladrin®), a gene therapy that uses a non-replicating adenoviral vector to deliver the gene for IFN-α2b (Martini et al. 2023).

However, the clinical development path for NMIBC has proceeded under the implicit assumption that IFN-α is the optimal interferon for this indication. The type I-IFN family includes both multiple IFN-α subtypes and a single IFN-β, all of which signal through the same receptor complex. Yet, the possibility that these interferons possess differential potencies in the specific context of BLCA has not been rigorously investigated. This represents a critical knowledge gap. If significant differences in potency exist, the field may be pursuing a suboptimal therapeutic strategy.

Interferons (IFNs) are class II cytokines of the innate immune system and are grouped into type I-, II- and III-IFNs (Lazear et al. 2019). All IFNs activate the JAK/STAT pathway but bind distinct receptor subunits (RS): type I-IFNs to IFNAR1/2 and type III-IFNs to IFNLR1/IL10RB. Receptor engagement induces dimerisation and Janus kinase (JAK)-mediated phosphorylation. Type I- and III-IFNs primarily activate signal transducers and activators of transcription 1 and 2 (STAT1, STAT2) (Murphy et al. 2018). Together with the IFN-regulatory factor 9 (IRF9), the heterodimer of STAT1 and STAT2 forms the IFN-stimulated gene factor 3 (ISGF3), which translocates to the nucleus and binds to the IFN-sensitive response element (ISRE) to induce interferon-stimulated genes (ISGs) (Mowen & David, 1998). Over 300 ISGs have been identified (Schoggins, 2019). This study focused on six ISGs expressed in the analysed BLCA cells: IFN-induced protein with tetratricopeptide repeats 1 and 2 (*IFIT1*, *IFIT2*), *IRF9*, IFN-stimulated gene 15 (*ISG15*), IFN-induced GTP-binding protein (*MX1*) and IFN-induced protein 44 (*IFI44*). These ISGs mediate antiviral, antiproliferative and immunomodulatory functions. The last two being most relevant for this work.

Here, we performed the first direct, head-to-head comparison of the effects IFN-α2, -β and -λ1 on BLCA cell lines. Our goal was to quantify their relative abilities to activate the canonical JAK/STAT signaling pathway, induce the expression of key ISGs and elicit anti-proliferative and pro-apoptotic effects. By providing a direct comparison of potency, we aimed to generate the basic fundamental data necessary to either validate the current focus on IFN-α or provide a compelling rationale for re-evaluating which IFN holds the most promise for the treatment of NMIBC.

In detail, to evaluate this functional importance of IFNs on activation of the innate immune system in BLCA, we analysed the anti-tumour effects of the type I-interferons IFN-α2 and IFN-β as well as of the type III-interferon IFN-λ1 on the BLCA cell lines SW780 and RT4. We analysed the expression of the IFN RS IFNAR1, IFNAR2, IFNLR1 and IL10RB and a BLCA-specific set of ISGs. Furthermore, we studied the induction of the JAK/STAT signalling pathway in SW780 and RT4 cells, as well as the induction of ISRE and apoptosis in SW780 cells.

## Materials and methods

*Evaluation of TCGA data.* Data from The Cancer Genome Atlas (TCGA) project was used for assessment of the expression of IFN RS in BLCA. As part of this project, 412 samples from muscle invasive bladder Cancer (MIBC) and high-grade BLCA, that were chemotherapy-naive, were analysed (Weinstein et al. 2014). The GEPIA (Gene Expression Profiling Interactive Analysis) website visualises TCGA data and data from the Genotype-Tissue Expression (GTEx) project (404 BLCA samples) (Tang et al. 2017). Gene expression analysis of the RS *IFNAR1*, *IFNAR2*, *IL10RB*, and *IFNLR1* was conducted by comparing BLCA tissues with normal tissues, followed by correlation analysis of these genes within the BLCA samples. The expression data is given in the unit transcript per kilobase million (TPM). The results of the assessment of the gene expression data of the RS *IFNAR1*, *IFNAR2*, *IL10RB* and *IFNLR1* were given in the unit log_2_(TPM + 1). The significance level of the p-value was set at 0.01 (Tang et al. 2017).

*COSMIC database.* Data from the Catalogue Of Somatic Mutations In Cancer (COSMIC) was used to see if mutations could be detected in STAT1, STAT2 and IRF9 genes in RT4 and SW780 cells. Half of the data is sourced from supplementary tables in published studies, while the other half comes from genome consortia e.g. TCGA (Sondka et al. 2024).

*cBioPortal for Cancer Genomics.* This web-based platform visualises and analyses multidimensional cancer genomics data. The portal reduces molecular profile data from e. g. cancer tissues to e.g. genetic and gene expression events (Cerami et al. 2012; Gao et al. 2013). It was used to determine whether mutations are present in STAT1, STAT2 and IRF9 in BLCA tissue.

*ISGs*. For assessment of IFN action, ISGs were selected that are expressed in the BLCA. For this purpose, a data search was carried out in The Human Protein Atlas (Uhlén et al. 2005, proteinatlas.org).

*Cell culture*. SW780 cells (ATCC, CRL-2169) were cultured in Dulbecco’s Modified Eagle’s Medium (DMEM) complete with 4.5 g/l glucose and 10% fetal bovine serum (FBS, all Thermo Fisher Scientific). RT4 cells (DSMZ, ACC 412) were cultured in DMEM (4.5 g/l glucose) with 10% FBS and 5% essential amino acids (all Thermo Fisher Scientific). Both cell lines were incubated at 37 °C and 5% CO_2_ in a humid environment.

*Cell stimulation*. SW780 and RT4 cells were seeded in 96-well microplates and grown to 40–50% density. They were stimulated with different concentrations ranging between 2.5 and 250,000 pg/ml IFN-α2, -β and -λ1 (Biozol). The IFN were dissolved in DMEM.

*RNA and cDNA preparation.* RNA was isolated using the Direct-zol™ RNA MiniPrep kit (Zymo Research). RNA was aliquoted and frozen at -80 °C. cDNA was made from 500 ng RNA using SuperScript™ II Reverse Transcriptase (Thermo Fisher Scientific).

*Quantitative PCR (qPCR)*. qPCR was performed on a LightCycler 480 (Roche Diagnostics) using GoTaq® qPCR Master Mix (Promega). The following Taqman assays (Thermo Fisher Scientific) were used: HPRT1 (Hs02800695_m1), IFI44 (Hs00197427_m1), IFIT1 (Hs01675197_m1), IFIT2 (Hs00533665_m1), IFNAR1 (Hs01066118_m1), IFNAR2 (Hs01022060_m1), IFNLR1 (Hs00417120_m1), IL10RB (Hs00175123_m1), IRF9 (Hs00196051_m1), ISG15 (Hs00192713_m1), MX1 (Hs00895608_m1), TBP (Hs00427620_m1). The geometric mean calculated from the reference genes TBP and HPRT1 was used for normalisation.

*Western Blot (WB).* SW780 and RT4 cells were seeded in six-well microplates and grown to 70% density. Cells were stimulated with 25.000 pg/ml IFN-α2, -β and -λ1. After 4 h and 24 h cells were lysed. For this purpose, 200 µl of extraction buffer was added to each well and resuspended. The cells were then incubated on ice for 30 min, placed in an ultrasonic bath (frequency 35 kHz) for 10 min, shaken for 30 min at 4 °C and 2000 rpm and centrifuged for 20 min at 4 °C and 16,100 × g. Then the supernatants were diluted and proceeded to 4–12% NuPAGE Bis–Tris-gel electrophoresis (Thermo Fisher Scientific). The gel was blotted using the iBlot Dry Blotting System and iBlot Gel transfer Nitrocellulose stacks (Thermo Fisher Scientific). The following primary and secondary antibodies were used: Histon H3, STAT1, pSTAT1, STAT2, pSTAT2, IRF9 and anti-rabbit IgG, HRP-linked antibody (Supplementary Material S1). WB data were quantified densitometrically using the Image Studio Lite Ver 5.2 (LI-COR). The pSTAT1 and STAT1 protein fraction were first normalised to the respective histone H3 fraction. Then, a ratio was formed from the normalised pSTAT1 and STAT1 phosphoprotein content to calculate the relative protein content: (pSTAT1/Histone H3) / (STAT1/Histone H3).

*Reporter Assay.* Dual luciferase assay was performed with the Nano-Glo® Dual-Luciferase® Reporter Assay System (Promega). All reagents were prepared according to kit instructions. SW780 cells were seeded in 96-well microplates and grown to 60–70% density. The transfection agent FuGENE® HD (Promega) and the vectors pEGFP-N1 (Clontech Laboratories), pGL4.53[luc2/PGK] or pNL[NlucP/ISRE/Hygro] (both Promega) were added in a FuGENE® HD:DNA ratio of 4:1. The pEGFP-N1-vector was used for transfection control and the pGL4.53[luc2/PGK]-vector was used to normalise the ISRE luciferase signal. After the transfection over 24 h cells were stimulated with 25.000 pg/ml IFN-α2, -β, -γ and -λ1. Another 24 h later dual luciferase assay was performed.

*Caspase-3/7 Activity.* SW780 cells were seeded in 96-well microplates and grown to 15–25%. Cell culture supernatant was removed after 24 h and the cells were stimulated with 25.000 pg/ml IFN-α2, -β and -λ1, the apoptosis inducer cycloheximide as a positive control (p. c.) and with pure medium as a negative control (n. c.) over 24 h and 72 h. The caspase-3/7 assay was performed up to 72 h after stimulation. The IncuCyte® Apoptosis Assay with the IncuCyte® Caspase-3/7 Green Apoptosis Reagent (all Sartorius) was used to detect the activation of caspases 3 and 7 in SW780 cells. In addition, the IncuCyte® Nuclight Rapid Red Dye for live-cell nuclear labelling (Sartorius) was added to visualise nuclei. The Incucyte device calculated the red object count per image, which can be used as a measure for the proliferation, and the confluence [%] for evaluation. The cell nuclei of SW780 cells (red objects) and SW780 cells in which caspases-3/7 were activated (green objects) were counted. Then the ratio of green to red objects [%] was calculated.

*WST-1 viability measurement.* This endpoint determination was performed after 24 h stimulation. 10 µl water soluble tetrazolinium (WST-1, Roche Applied Science) were added to the wells and the absorbance was measured at 450 nm with a reference of 620 nm by the Mithras LB 940 microplate reader (Berthold Technologies) after 60 min (SW780) and 120 min (RT4).

*Statistical analysis*. In the WST-1 measurement the values of samples after stimulation were related to samples without stimulation, i.e. n. c.: (100 x (mean value_stimulation_-mean value _n.c_./mean value_n.c_.). The Mann–Whitney-U-test was used for the statistical calculation of the significance level of differences between independent samples. The test was performed with GraphPad Prism Version 9.0 (GraphPad Software). A p-value of < 0.05 indicated a significant result (fixed significance level). The standard deviation (SD) was calculated for multiple determinations. The Pearson correlation coefficient (PCC) was used to analyse potential correlations between the gene expression of the RS *IFNAR1*, *IFNAR2*, *IL10RB* and *IFNLR1*.

## Results

### Expression analyses of IFN receptor subunits in BLCA tissues and cell lines

The expression of the RS of the type I- and III-IFN receptors in BLCA cells is the requirement for a successful response to stimulation of the cells with type I- and III-IFN. Therefore, database search and expression analysis by qPCR was done. The database search was intended to serve as a comparison between the tissue data from the TCGA project and own expression data collected in the BLCA cell lines.

**Fig. 1:**
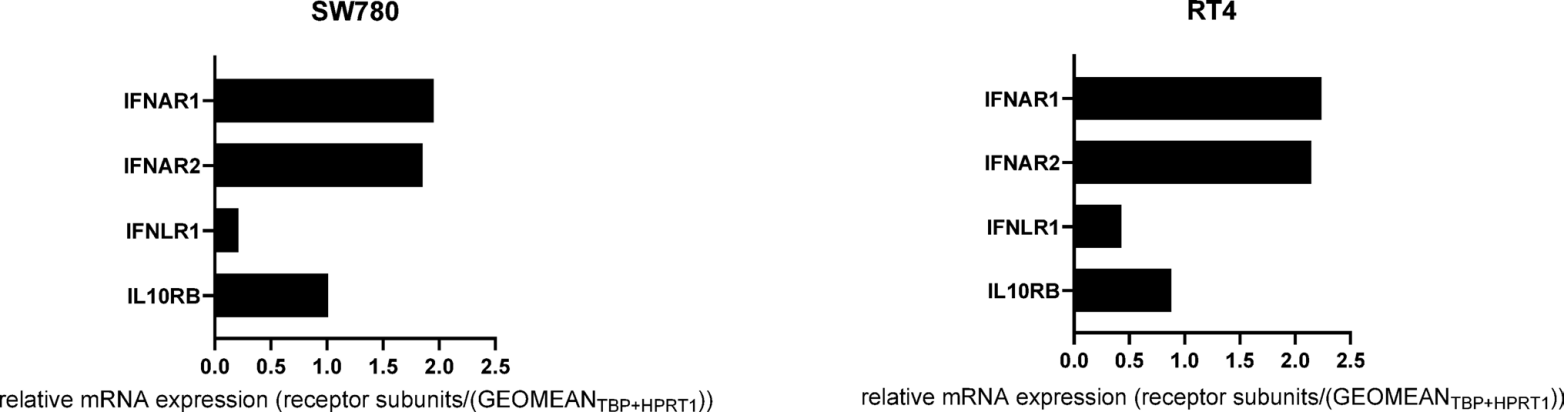
Relative mRNA expression of the receptor subunits *IFNAR1, IFNAR2, IFNLR1* and *IL10RB* in SW780 and RT4 cells. RNA was isolated from the individual cell lines, reverse-transcribed into cDNA and then analysed for expression of the IFN RS by qPCR. The expression levels of the individual RS were normalised to the geometric mean of the reference genes TBP and HPRT1

The search in the TCGA data confirmed that the RS *IFNAR1*, *IFNAR2*, *IFNLR1* and *IL10RB* are expressed in normal and in BLCA tissue (Supplementary Material S2). Our own qPCR experiments confirmed that the RS were expressed in many different BLCA and non-malignant cell lines (Supplementary Material S3). Among all analysed cell lines, the BLCA cell lines SW780 and RT4 expressed the RS most strongly. The expression of *IFNAR1* and *IFNAR2* was higher compared to *IFNLR1* and *IL10RB*, with the expression of *IFNLR1* being the lowest (Fig. 1). Correlation analyses showed that there was a strong positive association between the subunits of the type I-IFN receptor *IFNAR1* and *IFNAR2* (R = 0.65, *p* < 0.001), but only a weak, but also significant positive association between the subunits of the type III-IFN receptor *IFNLR1* and *IL10RB* (R = 0.15, *p* = 0.0022) (Supplementary Material S4).

### No short-term inhibition of viability after IFN stimulation of BLCA cells

**Fig. 2:**
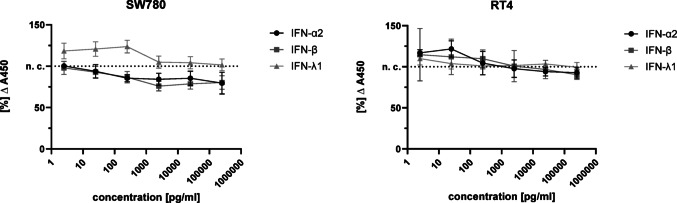
Influence of stimulation with 2.5–250,000 pg/ml IFN-α2, -β and -λ1 over 24 h on the cell viability of the RT4 and SW790 cell lines. The viability measurements were performed for 60 min for the RT4 cells and 120 min after addition of WST-1 for the SW780 cells. The respective MW of the measurements after stimulation were related to the negative control to calculate the percentage change. The significance was then calculated using the Mann–Whitney-U test,p > 0.05 (n = 9, 3 trials with triplicates), ± SD

The WST-1 assay was used to analyse the influence of a stimulation with 2.5–250,000 pg/ml IFN-α2, -β and -λ1 on the viability of RT4 and SW780 cells (Fig. 2). Overall, no significant inhibition of viability after 24 h stimulation with IFN was recognisable in both cell lines over the entire concentration range. Nevertheless, a reduction of viability with increasing concentration of IFN was recognisable by trend (Fig. 2).

### Long-term induction of apoptosis after IFN stimulation of BLCA cells

In order to find out whether stimulation with IFN at a later time point had an influence on BLCA cell proliferation, the caspase-3/7 assay was performed in the SW780 cell line. The island-like cell growth of the RT4 cells did not allow a reliable analysis of apoptotic cells. Induction of apoptosis was assessed up to 72 h after stimulation of SW780 cells with 25,000 pg/ml IFN-α2, -β and -λ1. SW780 cells were also stimulated with the apoptosis inducer cycloheximide as a p. c. and with pure medium as a n. c.. The apoptosis induction (green object count / red object count [%]) of the IFN- and cycloheximide stimulated cells compared to the non-stimulated cells (n. c.) was analysed and the significance was calculated. For example, after 24 h, apoptosis induction was significantly higher after stimulation with IFN-α2 compared to n. c. (**), but not with IFN- λ1 compared to n. c. (ns).

**Fig. 3:**
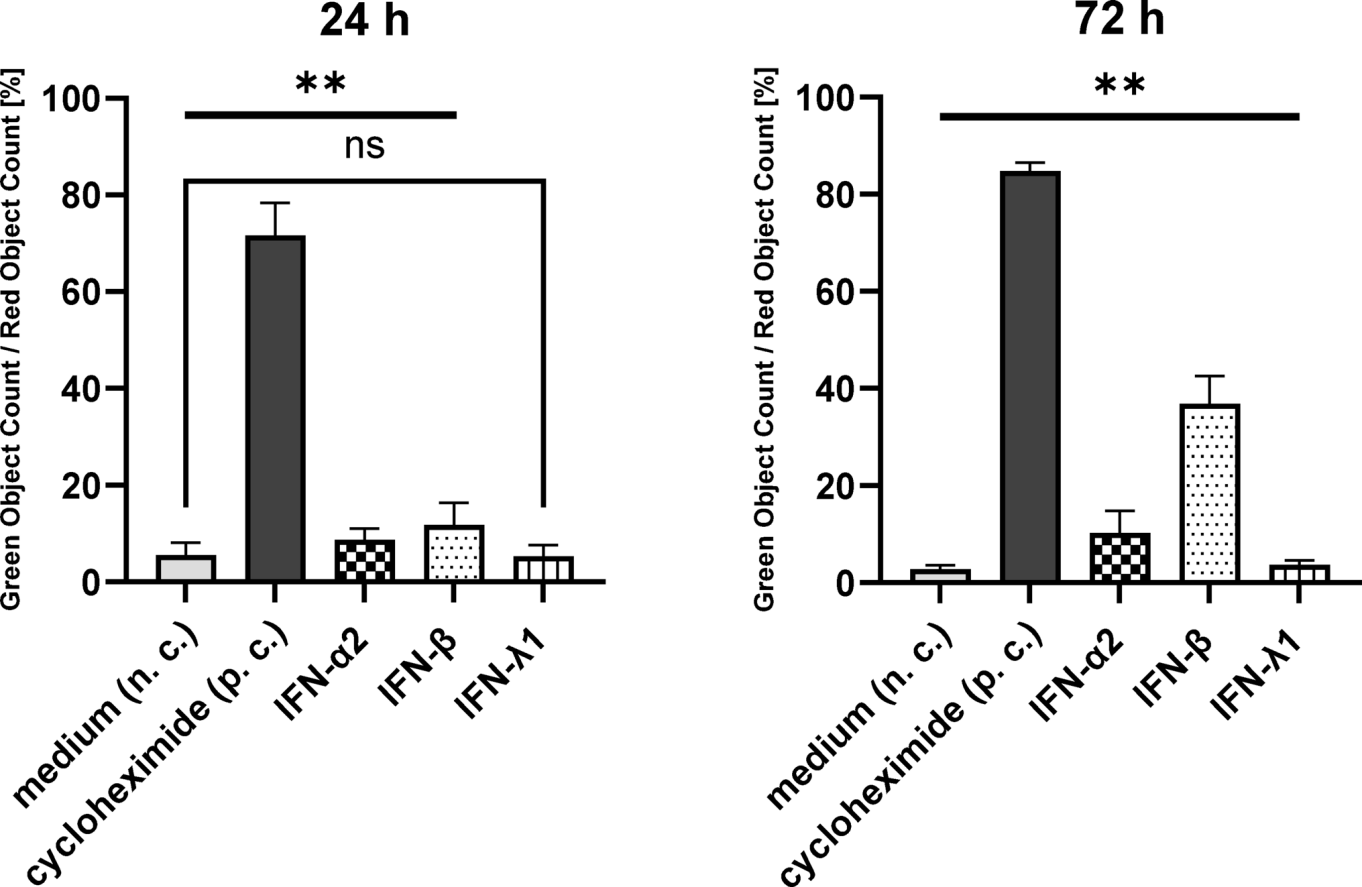
Caspase-3/7 assay for the detection of apoptosis by stimulation of SW780 cells with 25,000 pg/ml IFN-α2, -β, -λ1 and cycloheximide for 24 h and 72 h. Green Object Count / Red Object Count [%] to calculate the proportion of apoptotic cells (green) to cell nuclei (red), ± SD. Cells stimulated with IFN and cycloheximide were compared with non-stimulated cells (n. c.). **: significant, ns: not significant

After 24 h no significant changes in confluence and red object count induced by IFN stimulation were detected (Fig. 3, confluence not shown). There were more apoptotic cells after stimulation with IFN-β (11.8%) than after stimulation with IFN-α2 (8.7%) and -λ1 (5.3%, Fig. 3). The number of apoptotic cells increased 72 h after stimulation with IFN-α2 (to 10.3%) and very clear with IFN-β (to 26.8%) (Fig. 3).

### IFN-β stimulated ISGs most strongly

For the evaluation of IFN activity, ISGs expressed at RNA level in BLCA were identified. To this end, data were obtained through a search in The Human Protein Atlas (Uhlén et al. 2005; proteinatlas.org). The following six ISGs were found to be strongly expressed in SW780 and RT4 cells*: IFI44, IFIT1, IFIT2, IRF9, ISG15* and *MX1*.

**Fig. 4:**
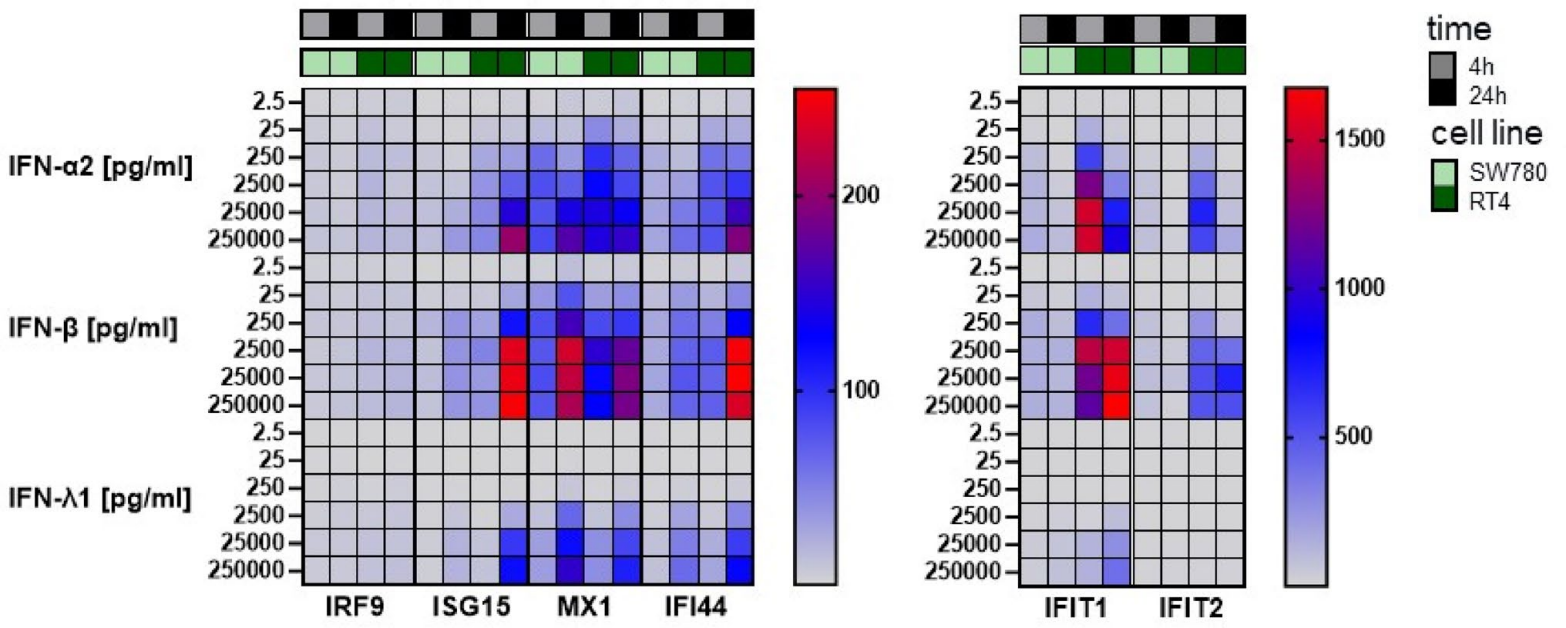
Heatmap of n-fold expression of the ISGs *IFIT1, IFIT2, IRF9, ISG15, MX1* and *IFI44* after 4 h and 24 h in the BLCA cell lines SW780 and RT4. The cells were stimulated with 2.5–250,000 pg/ml IFN-α2, -β and -λ1. The stimulated samples were normalised to unstimulated samples (*n* = 3). RNA was isolated from the individual cell lines, transcribed into cDNA and then expression analysis was performed by qPCR. The expression levels of the individual ISGs were normalised to the geometric mean of the reference genes TBP and HPRT1. The mean value was then calculated from *n* = 3

To demonstrate the induction of ISGs, SW780 and RT4 cells were stimulated with increasing concentrations (2.5 to 250,000 pg/ml) of IFN-α2, IFN-β and IFN-λ1. Expression analysis of six selected ISGs was subsequently performed using qPCR. In both cell lines it was observed that there was a concentration-dependent induction of the ISGs (Fig. 4). The ISGs were induced more strongly in the RT4 cell line than in the SW780 cell line. IFN-α2 and -β induced ISGs more strongly than IFN-λ1. In the SW780 cell line, n-fold expression of *IFIT1* was the highest after 4 h of stimulation with e.g. 250,000 pg/ml IFN-α2 (157.4), IFN-β (148.1) and IFN-λ1 (33.9). After 24 h of stimulation with 250,000 pg/ml of each IFN n-fold expression of *MX1* was the highest (IFN-α2: 169.0, IFN-β: 212.9, IFN-λ1: 151.7) followed by *IFIT1* (IFN-α2: 92.0, IFN-β: 130.8, IFN-λ1: 76.5). In the RT4 cell line, n-fold expression of *IFIT1* was clearly highest after 4 h (IFN-α2: 1525.3, IFN-β: 1137.6, IFN-λ1: 135.9) and 24 h (IFN-α2: 912.9, IFN-β: 1675.6, IFN-λ1: 396.5) of stimulation with 250,000 pg/ml of each IFN. *IFIT2* was also strongly expressed, even though significantly lower than *IFIT1* (after 4 h: IFN-α2: 559.8, IFN-β: 504.4, IFN-λ1: 4.9; after 24 h: IFN-α2: 164.9, IFN-β: 522.1, IFN-λ1: 13.3).

*IRF9* expression was also induced by IFN stimulation in both cell lines, but only at such low level, that it is not clearly visible in Fig. 4. After 24 h stimulation with 250,000 pg/ml each, IRF9 expression was more strongly stimulated in RT4 cells (IFN-α2: 17.1, IFN-β: 19.1, IFN-λ1: 13.0) compared to SW70 cells (IFN-α2: 10.4, IFN-β: 10.5, IFN-λ1: 7.5). Overall, type I-IFNs induced ISGs more strongly than type III-IFNs and IFN-β induced more ISGs than IFN-α2 (Fig. 4).

### IFN-β activates JAK/STAT signalling pathway most strongly

**Fig. 5:**
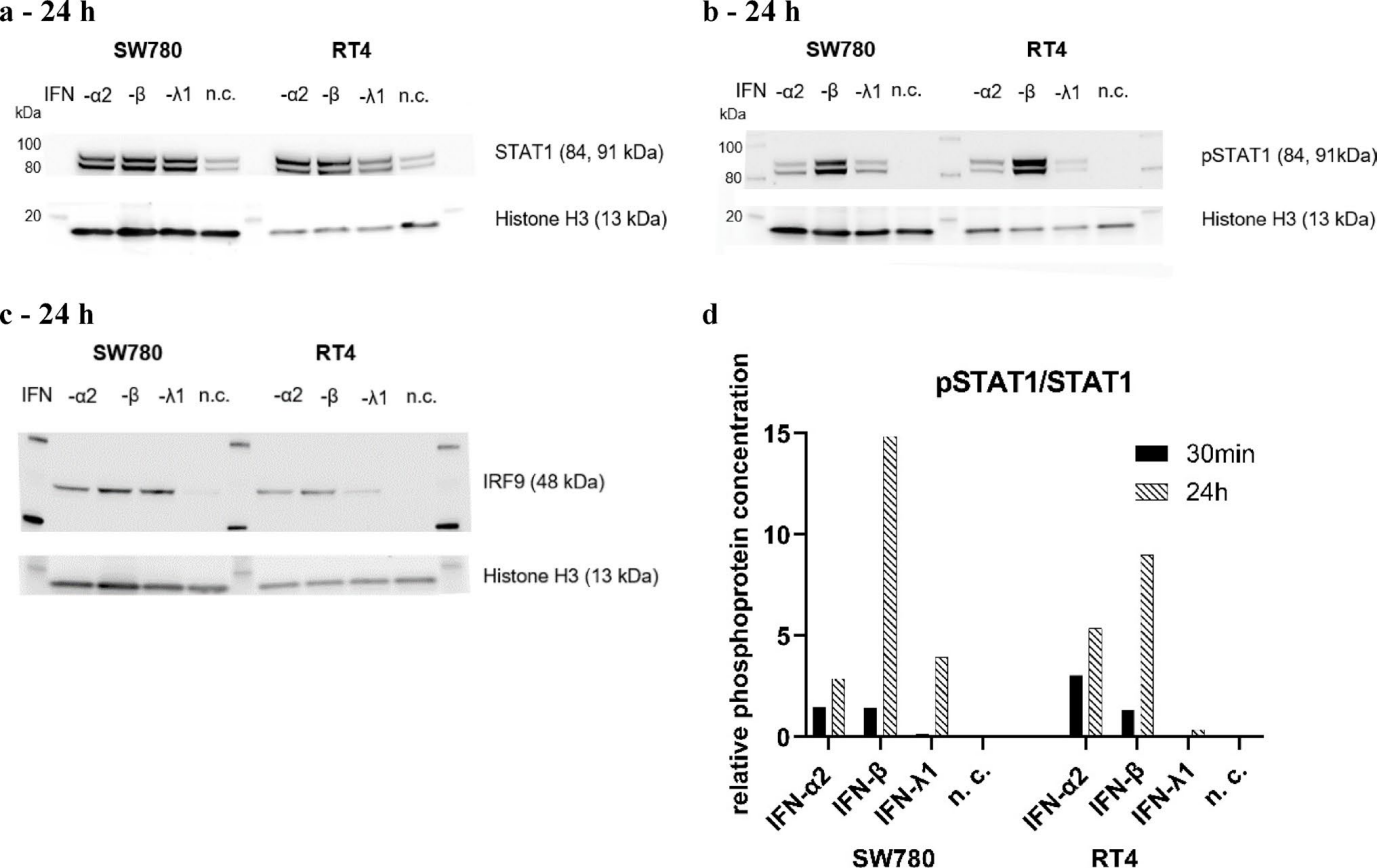
Example western blot analysis of STAT1 and pSTAT1 in SW780 and RT4 cells after 24 h with calculation of the relative phosphoprotein concentration. Additional western blot analysis of IRF9 was done after 24 h. SW780 and RT4 cells were harvested after 24 h stimulation with 25,000 pg/ml IFN-α2, -β and -λ1, lysed and Western blot labeled with antibodies against **a** STAT1, **b** pSTAT1, **c** IRF9 and (a, b, c) histone H3 and detected via HRP-coupled secondary antibodies. The protein bands were analysed densitometrically and the pSTAT1 and STAT1 protein fraction was normalised to the histone H3 fraction. A ratio was then formed from the normalised pSTAT1 and STAT1 phosphoprotein content to calculate the relative protein content: **d** (pSTAT1/Histone H3) / (STAT1/Histone H3)

After stimulation of SW780 and RT4 cells with 25,000 pg/ml IFN-α2, -β and -λ1 for 30 min (not shown) and 24 h, WB analyses were performed to the induction and activation of STAT1 and STAT2 by phosphorylation as well as of IRF9 (Fig. 5). The detection of STAT1 and STAT2 in the WB reflected total STAT1 and STAT2, regardless of phosphorylation status, whereas the detection of pSTAT1 and pSTAT2 served as confirmation of IFN activation of the signalling pathway by stimulation.

**Fig. 6:**
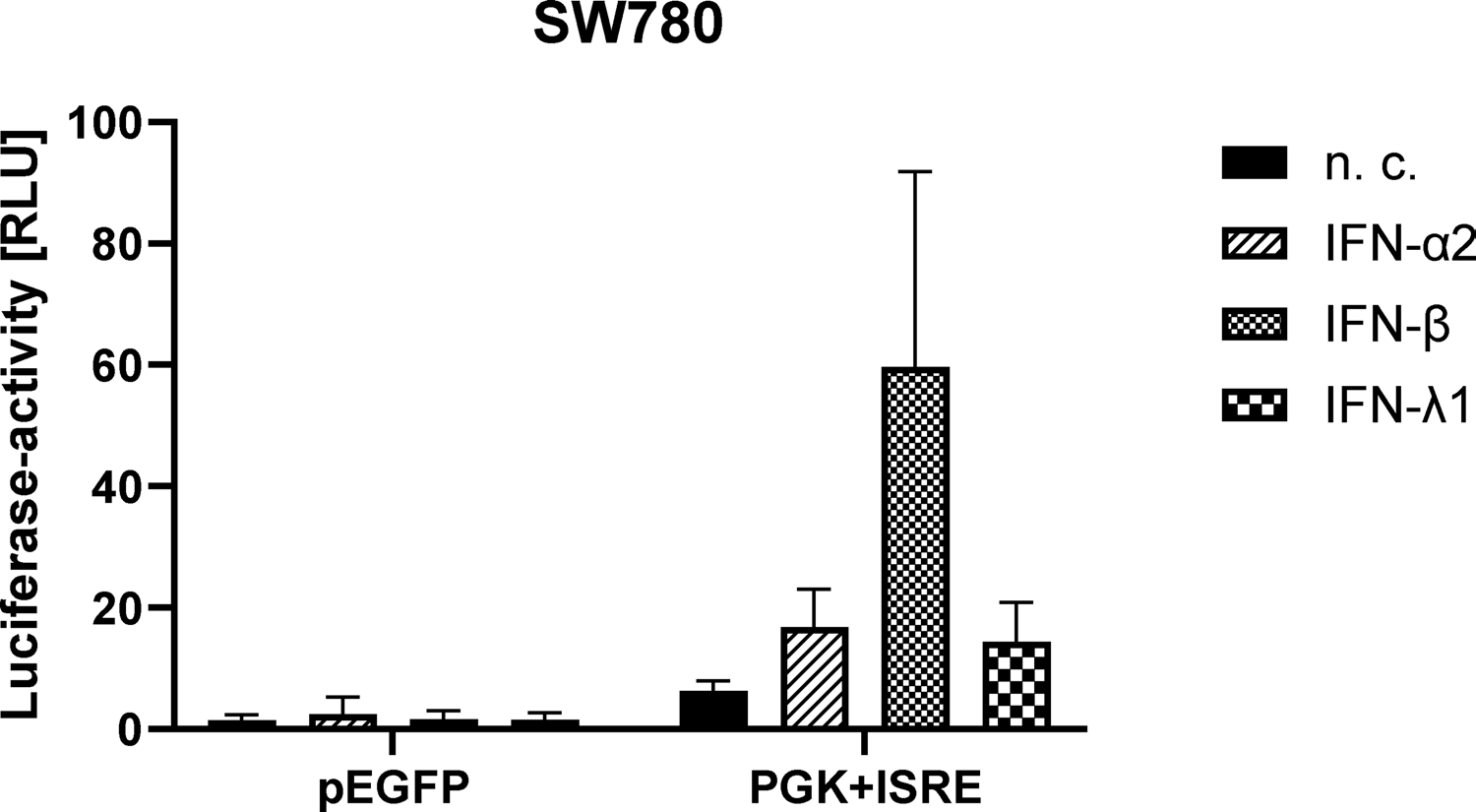
Luciferase reporter assay to measure the activation of the IFN-sensitive response element (ISRE). Luciferase activity was measured in the SW780 cell line after 24 h stimulation with 25,000 pg/ml IFN-α2, -β and-λ1 (*n* = 4, SD). Relative luciferase activity was calculated by normalising NanoLuc® luciferase activity to Firefly luciferase activity. The pEGFP vector served only as a control for transfection efficiency, but did not induce ISRE

After 30 min and 24 h of stimulation STAT1 was detectable in all stimulated cells and in the n. c. (Fig. 5a). In contrast, pSTAT1 was only detectable in IFN-stimulated samples, especially in the samples stimulated with IFN-α2 and IFN-β (Fig. 6b). In the samples stimulated with IFN-λ1 weak bands of pSTAT1 were recognisable in both cell lines (Fig. 5b). pSTAT1 was not detectable in the n. c. after 30 min (data not shown) and 24 h (Fig. 5b). According to the semi-quantitative densitometrical evaluation of pSTAT1/STAT1, the relative phosphoprotein concentration was highest in the SW780 and RT4 cell lines after 24 h and after stimulation with IFN-β (Fig. 5d). The WB analyses for STAT2 and pSTAT2 showed very similar results to those for STAT1 and pSTAT1 (data not shown). Detection of IRF9 in SW780 and RT4 cells was only possible after 24 h of stimulation. IRF9 is an ISG and therefore it is possible that IRF9 was only upregulated after 24 h in all stimulated samples (Fig. 5c).

### ISRE was activated by type I- and III-IFNs

The luciferase reporter assay was used to measure the activation of the responsive element ISRE in the SW780 cell line. The RT4 cell line exhibited island-like cell growth, which was why transfection and evaluation of the transfection efficiency was not possible in this cell line. Similar to the caspase-3/7 assay, this experiment was only carried out with the SW780 cell line. Using WB, it was shown at the protein level that IFN stimulation upregulated IRF9 expression (Fig. 5c). As already mentioned, pSTAT1, pSTAT2 and IRF9 form the ISGF3 complex, which translocates into the cell nucleus and binds to ISRE. Using the luciferase reporter assay, it was shown that ISRE was activated via IRF9 by IFN-α2, -β and -λ1. IFN-α2 and -λ1 activated ISRE at a similar level. IFN-β activated ISRE most strongly (Fig. 6). The pEGFP vector served only as a control for transfection efficiency, but did not induce ISRE (i.e. no luciferase-activity). According to microscopic inspection the transfection with the pEGFP vector was successful, which allowed the conclusion that the transfection with the other vectors was also effective.

## Discussion

### Expression of IFN receptor subunits in BLCA tissues and cells as prerequisite for IFN stimulation

The GEPIA platform (Tang et al. 2017) allowed the assessment of the TCGA data (Weinstein et al. 2014) regarding the basic expression of the RS in BLCA tissue (red, Supplementary Material S2) and normal tissue (grey, Supplementary Material S2). All four RS were expressed in BLCA and normal tissue. Similar behaviour was observed in our own qPCR analyses with the BLCA cells SW70 and RT4. Here, it could be shown that the RS were expressed in the cells too (Fig. 1). Therefore, these cell lines were suitable as a model for the following stimulation experiments. The analysis of TCGA data revealed that the expression of the RS of type I-IFN-receptor *IFNAR1* and *IFNAR2* strongly correlated in BLCA tissues (Supplementary Material S4). We detected an analogous behaviour in the analysed BLCA cell lines (Fig. 1).

Correlation analysis of the RS revealed only a very weak association between IL10RB and IFNLR1, although both form the type III-IFN receptor (Supplementary Material S4). In our SW780 and RT4 expression data, IL10RB and IFNLR1 showed no correlation (data not shown). While IL10RB levels were elevated in both cell lines, IFNLR1 expression remained low (Fig. 1), indicating that IFNLR1 is the limiting factor for effective type III-IFN signalling. Similar observations have been reported in hepatocytes, where restricted IFNLR1 expression reduced antiviral responses (Novotny et al. 2024). Nonetheless, the data confirmed that IFNs can bind to these cells, enabling subsequent stimulation experiments.

### Type I- and III-IFN induced significantly apoptosis in long-term stimulation

To observe changes in cell viability, the WST-1 assay was performed with RT4 and SW780 cells after 24 h (Fig. 2). In addition, the caspase-3/7 assay was used to analyse possible apoptosis induction from 24 h up to 72 h after IFN stimulation. An early restriction of the viability of RT4 and SW780 cells under IFN stimulation would have a negative effect on the cytokine production by the cells. However, the evaluation of the WST-1 assay after short-term stimulation showed only a slight change in viability in either cell line after 24 h (Fig. 2). Type I- and III-IFNs can trigger apoptosis in various tumour cell line models (Chawla-Sarkar et al. 2003; Li et al. 2008; Steen & Gamero 2010). This could be confirmed by our own data: After 24 h IFN-α2 and -β induced significantly more apoptosis in the SW780 cell line compared to the negative control (Fig. 3). After 72 h, IFN-λ1 induced apoptosis significantly more than the negative control, approximately 1.3-fold higher (Fig. 3). Comparing only the effects of IFN stimulation after 24 h and 72 h, it was noticeable that the apoptosis rate induced by IFN-α2 remained approximately the same after 24 h and 72 h. However, the apoptosis rate induced by IFN-β increased by approximately 2.4-fold over the 24 h and 72 h periods (Fig. 3). The measurements of the caspase-3/7 assay showed that IFN induced apoptosis in the short term (24 h), but especially in the long term (72 h).

### Activation by type I- and III-IFN was mediated via ISRE

Type I-IFNs IFN-α2 and IFN-β are known to activate the induction of ISRE (Darnell et al. 1994). Activation of ISRE is a prerequisite for ISG induction, which has been shown to be upregulated by stimulation with IFN-α2, -β and -λ1 (Fig. 2). For type III-IFNs, it is assumed that they behave similarly to type I-IFNs and also switch on ISRE (Ye et al. 2019). The performed luciferase reporter assay confirmed that assumption that IFN-α2, -β and -λ1 act via the responsive element ISRE (Fig. 6). IFN-β was also the most active here. To date, there are no comparable reporter gene assays in other solid tumours that can serve as a comparison.

### IFN-β stimulated ISGs most strongly

Both IFN-α2 and IFN-β are type I-IFNs and activate the JAK/STAT signalling pathway through the same RS (Renauld 2003). IFN-β induced ISG expression more strongly than IFN-α2 in various cell line models. In melanoma cell lines, ISG expression was also induced more strongly by stimulation with IFN-β than with IFN-α2 (Leaman et al. 2003). Similar results were also shown in hepatocytes. There, all IFNs induced ISG expression, but to varying degrees: IFN-β > IFN-α2 > IFN-λ3 > IFN-λ1 > IFN-λ2 (Bolen et al. 2014). This may be due to the stronger binding affinity of IFN-β to IFNAR1/IFNAR2 compared to IFN-α, resulting in a more stable ternary complex (IFN-β/IFNAR1/IFNAR2) (Lavoie et al. 2011; Wittling et al., 2021). The stronger binding of IFN-β can induce higher ISG expression and a more pronounced antiproliferative state, so lower doses of IFN-β are sufficient to elicit antiproliferative activity (Schreiber 2017).It can be assumed that the main difference between IFN-β and IFN-α2 lies in the different binding affinity to the RS (Schreiber 2017). IFN-λ is expected to induce fewer ISGs, as its receptors are less abundantly expressed (Fig. 1) and are primarily restricted to cells of anatomical barriers (Lazear et al. 2 019).

### IFN-β activated JAK/STAT signalling pathway most powerful

STAT1, STAT2, their phosphorylated variants and IRF9 are key molecules of the JAK/STAT signalling pathway. The relative phosphoprotein concentration of pSTAT1/STAT1 in the in RT4 and SW780 cells was higher after stimulation with IFN-β than with IFN-α2 (Fig. 5d). Transferred to the BLCA tissues, the increased proportion of phosphorylated STATs indicated an increased ISG expression through stimulation with IFN-β. This was also confirmed by the ISG expression analysis (Fig. 4). As mentioned in the previous section, the increased ISG expression by IFN-β may also potentially result in strong antiproliferative, apoptotic and immunomodulatory properties against BLCA. Across both BLCA cell lines, IFN-β treatment resulted in a stronger and more sustained phosphorylation of STAT1, a more robust induction of key ISGs like IFIT1, IFIT2, IRF9, MX1, ISG15 and IFI44 a more potent pro-apoptotic effect compared to IFN-α or IFN-λ.IFN-β is the most potent inducer of the upstream interferon signaling cascade (pSTAT1 phosphorylation and ISG expression) in both cell lines tested.The relevance of these findings lies in their direct challenge to the established, IFN-α-centric paradigm in NMIBC therapy. The clinical and research landscape has been almost exclusively focused on IFN-α, culminating in the recent approval of an IFN-α-based gene therapy (Martini et al. 2023). Our data question the assumption that IFN-α is the optimal choice, suggesting instead that the field may have overlooked a more potent agent. While the superior potency of IFN-β has been noted in other malignancies, demonstrating this principle specifically in BLCA is a critical and non-incremental advance that provides the necessary evidence to reconsider the current therapeutic development strategies. The mechanistic basis for IFN-β’s superior potency is well-established, though not previously highlighted in the context of BLCA. Both IFN-α and IFN-β signal through the same IFNAR1/IFNAR2 receptor complex, but biophysical studies have shown that IFN-β possesses a significantly higher binding affinity for the IFNAR1 subunit (Lavoie et al. 2011). This leads to the formation of a more stable ternary signaling complex, resulting in the more sustained and robust STAT1 activation that we observed in our experiments. Our findings are therefore consistent with this established molecular model and provide the biological context for its relevance in BLCA.

Although our study was conducted in a 2D monoculture, our findings may have implications for the tumour microenvironment (TME). We have demonstrated that IFN-β potently can activate the cell-intrinsic molecular machinery required to initiate a favorable immune response. The upregulation of ISGs is a critical first step in remodeling the TME; many of these genes encode proteins that enhance antigen presentation on tumour cells or function as chemokines to recruit cytotoxic immune cells like NK cells and T-cells. For example, elevated expression of ISG15, which was induced in our model, is associated with increased immune infiltration in pan-cancer analyses (Wei et al. 2025) Therefore, while we could not directly measure immune infiltration, our results may provide a mechanistic rationale for investigating IFN-β as a strategy to convert the immunologically ‘cold’ bladder TME into a ‘hot’ state, thereby mimicking a key functional outcome of successful BCG therapy.

From a clinical standpoint, our study may provide two new treatment strategies. First, it suggests that a “second-generation” interferon gene therapy using a vector to deliver IFN-β could be more effective, based on the assumption that IFN-β gene therapy is safe and effective in other cancer types (Yoshida et al. 2004). Second, IFN-β should be investigated as a more potent adjuvant for BCG combination therapy, a strategy that could improve outcomes for BCG non-responders and enable the use of dose-sparing BCG regimens despite global shortages.

The combination of BCG with IFN-α2b has been used as a salvage approach in BCG-refractory NMIBC, based on the ability of IFN-α to potentiate BCG-induced Th1 immunity (O’Donnell, 2004; Correa et al. 2015), although its benefit over BCG alone remains uncertain in systematic reviews (Shepherd, 2017). The recent FDA approval of the IFN-α2b gene therapy nadofaragene firadenovec (Adstiladrin®) represents an important milestone in this field (Colbert et al. 2025; Lee 2023; Martini et al. 2023). In this context, our data suggest that IFN-β may exert distinct immunological effects compared to IFN-α (Westcott et al. 2015). These findings provide a rationale to further investigate IFN-β as a biologically differentiated partner for BCG in NMIBC.

The development of oncolytic viruses represents a promising new frontier for bladder cancer therapy, particularly for patients with treatment-resistant disease (Hu et al. 2021). The work of Zhang et al. (2010) provides a compelling mechanistic rationale for this approach, demonstrating that the downregulation of the type I-IFN receptor (IFNAR) in bladder cancer cells, which is a common mechanism of resistance to IFN-therapy, paradoxically sensitises these same cells to being killed by oncolytic viruses like VSV (Zhang et al. 2010). This inverse relationship is clinically significant, as it suggests that the IFNAR expression could serve as a predictive biomarker to stratify patients. Tumours with high IFNAR may be suited for IFN-based treatments, whereas those with low IFNAR would be candidates for oncolytic virus therapy.

### Limitations of the study

While our findings provide a strong rationale for prioritising IFN-β in future BLCA research, we must acknowledge several important limitations of the current study. Our experiments were conducted exclusively in a 2D monoculture system. This model, while useful for dissecting cell-intrinsic signalling pathways, cannot recapitulate the complex, three-dimensional architecture and cellular heterogeneity of the in vivo tumour microenvironment, which includes crucial interactions with immune cells, fibroblasts, and the extracellular matrix (Goulet et al. 2019). Therefore, our conclusions regarding the potential effects of IFN-β on the TME are hypotheses based on the observed induction of ISGs and require validation in more complex co-culture or in vivo models. The generalisability of our findings is constrained by the use of only two BLCA cell lines. Although RT4 and SW780 represent one tumour grade, they cannot capture the full molecular diversity of NMIBC. Further studies on a broader panel of cell lines are necessary to confirm that the superior potency of IFN-β is a universal phenomenon in this disease. Our dataset is incomplete due to technical challenges. We were unable to perform functional apoptosis and ISRE-luciferase reporter assays in the RT4 cell line due to its “island-like” growth pattern, which severely limits transduction efficiency. This growth pattern, while a technical hurdle, is biologically relevant as it mimics the papillary structures seen in vivo (Ewell et al. 2024). The dense, multi-layered nature of these colonies, combined with strong cell–cell junctions, creates a physical barrier that prevents efficient delivery of genetic material to the majority of cells within the island. Consequently, while the superior signalling effect of IFN-β was confirmed in both cell lines, its functional pro-apoptotic benefit was only demonstrated in SW780 cells.

## Conclusion

Stimulation with IFN-β induced a strong activation of the JAK/STAT signalling pathway in BLCA cell models. Firstly, this resulted in an upregulation of molecules of the JAK/STAT signalling pathway at the protein level. Secondly, IFN-β most strongly activated the responsive element ISRE and thirdly, it also induced ISG expression most strongly in the BLCA cell lines RT4 and SW780. Therefore, we concluded that IFN-β had the most potent effect. This has also been confirmed when measuring the induction of apoptosis, where IFN-β also had the strongest effect. This finding is based on the activation of the JAK/STAT signalling pathway. We could show that type I- and III-IFN, and especially IFN-β, had potent antiproliferative, immunomodulatory and apoptotic properties in the BLCA cell lines RT4 and SW780. In contrast type III-IFN IFN-λ1 showed a delayed effect, which was not as strong. Our study provides quantitative evidence that IFN-β induces stronger anti-tumour-associated responses in BLCA cells than IFN-α or IFN-λ under the conditions tested. These findings suggest that IFN-β, as a distinct agent from IFN-α, may warrant further investigation in future research and clinical development, for example as a potential adjuvant to BCG.

## Supplementary Information

Below is the link to the electronic supplementary material.

Supplementary Material 1

## Data Availability

The datasets generated during and/or analysed during the current study are available from the corresponding author on reasonable request.
