# Supplementary material for: Type I-interferon β induces a strong anti-tumour response in bladder cancer cells
Source: J Cancer Res Clin Oncol. 2026 Jan 8;152(1):35. doi: 10.1007/s00432-025-06409-1 (PMC12783495; doi:10.1007/s00432-025-06409-1)
Supplement: Supplementary file 1 — Supplementary Material 1 [file 432_2025_6409_MOESM1_ESM.docx]

# Supplementary Information

**Supplementary Material S1:** **Dilution of the primary monoclonal antibodies, size of the detected proteins and the secondary polyclonal antibody.** All antibodies of the JAK/STAT signaling pathway are from the IFN (Type I/III) Signaling Pathway Antibody Sampler Kit (Cell Signaling Technology).

| antibodies | kDa | dilution | clone |
| --- | --- | --- | --- |
| STAT1 | 84, 91 | 1:500 | D1K9Y |
| pSTAT1 | 84, 91 | 1:1000 | D4A7 |
| STAT2 | 97, 113 | 1:1000 | D9J7L |
| pSTAT2 | 97, 113 | 1:1000 | D3P2P |
| IRF9 | 48 | 1:1000 | D2T8M |
| Histon H3 | 15 | 1:1000 | D1H2 |
| Anti-rabbit IgG, HRP-linked antibody | -v | 1:5000 | - |


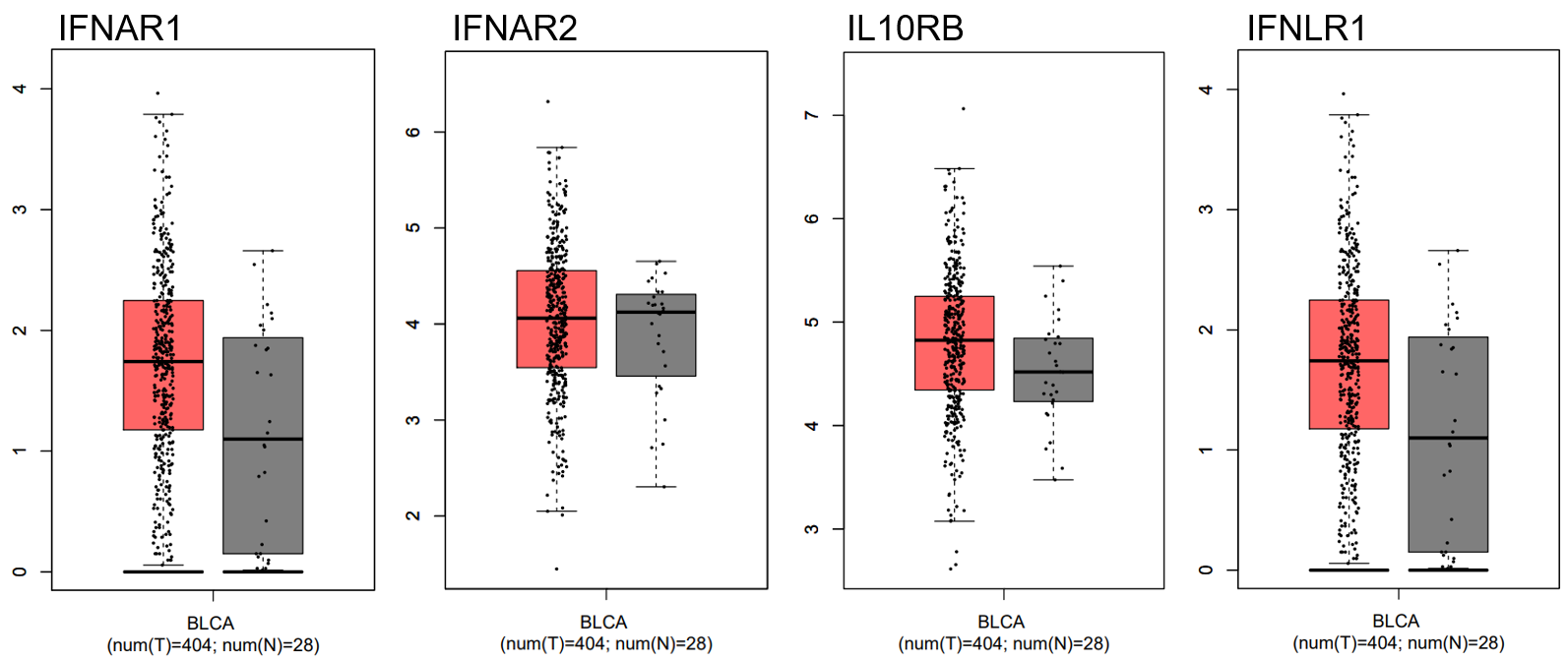


**Supplementary Material S2**: **Gene expression of the receptor subunits *IFNAR1, IFNAR2, IL10RB* and *IFNLR1* in direct comparison of BLCA (num(T)) and normal tissue (num(N)) shown as boxplots (log2 TPM+1).** Data are from the TCGA study. Tissue was obtained from MIBC and normal tissue adjacent to the tumour (Tang et al., 2017, GEPIA).

**Supplementary Material S3: Expression analysis of the receptor subunits *IFNAR1* and *IFNAR2* of the type I-IFN receptor and *IFNLR1* and *IL10RB* of the type III-IFN receptor** in all BLCA and non-malignant cell lines (NHDF (normal human dermal fibroblasts), NHBC (normal human bladder cells), and UROTSA (SV40 immortalized human urothelium cell line) available in the laboratory.

| **a** | **b** |
| --- | --- |
| 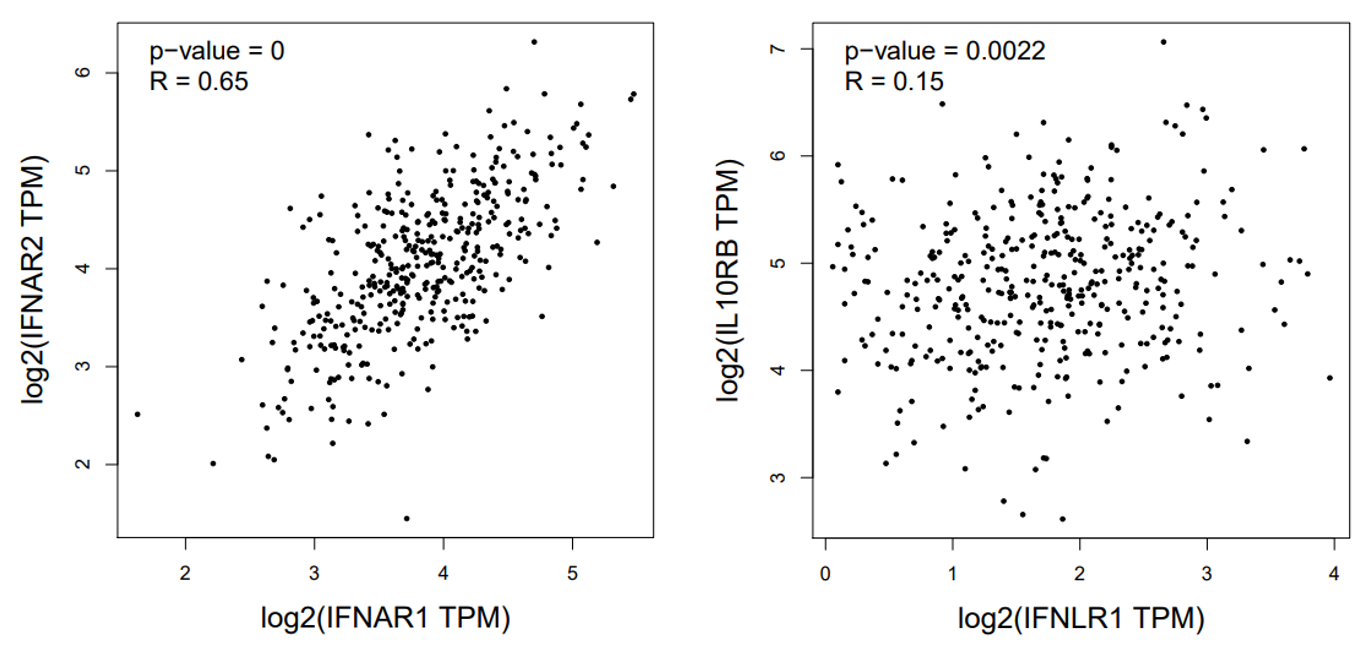  **Supplementary Material S4: Comparison of the correlation between the receptor subunits *IFNAR1, IFNAR2, IL10RB* and *IFNLR1*.** The data come from the TCGA study (Tang et al., 2017). The Pearson correlation coefficient (R) and the p-value were calculated. *INFAR1* and *INFAR2* (a) and *IFNLR1* and *IL10RB* (b) were compared (Tang et al., 2017, GEPIA). | |

| **a - STAT1 - 30 min** | **b - pSTAT1 - 30 min** |
| --- | --- |
| 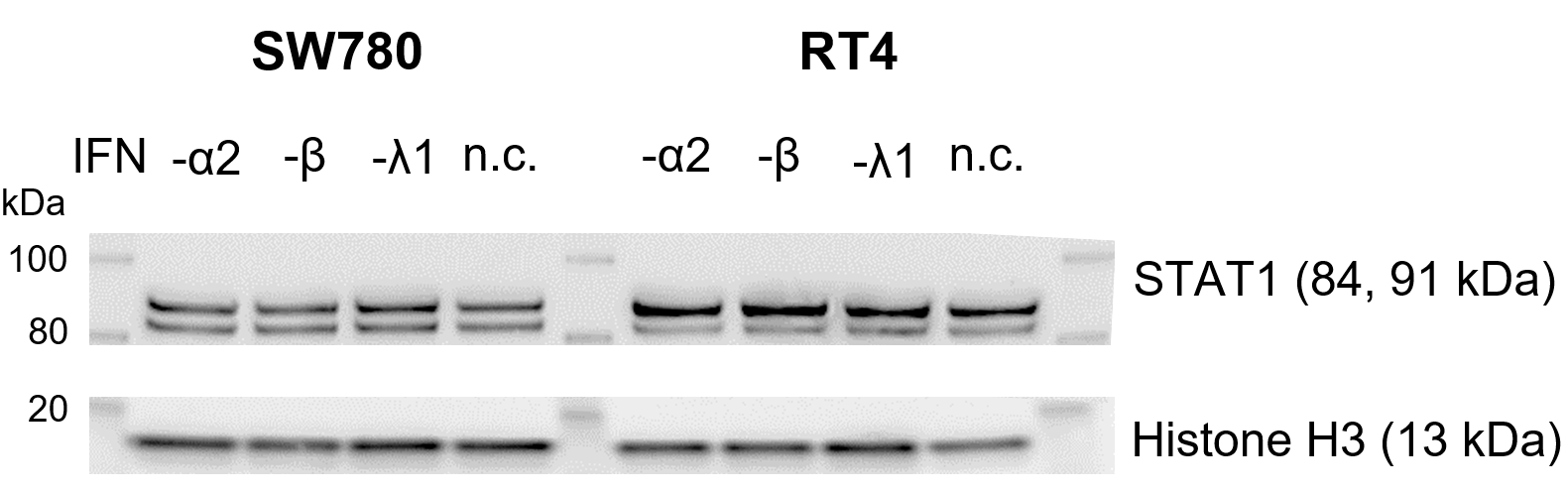 | 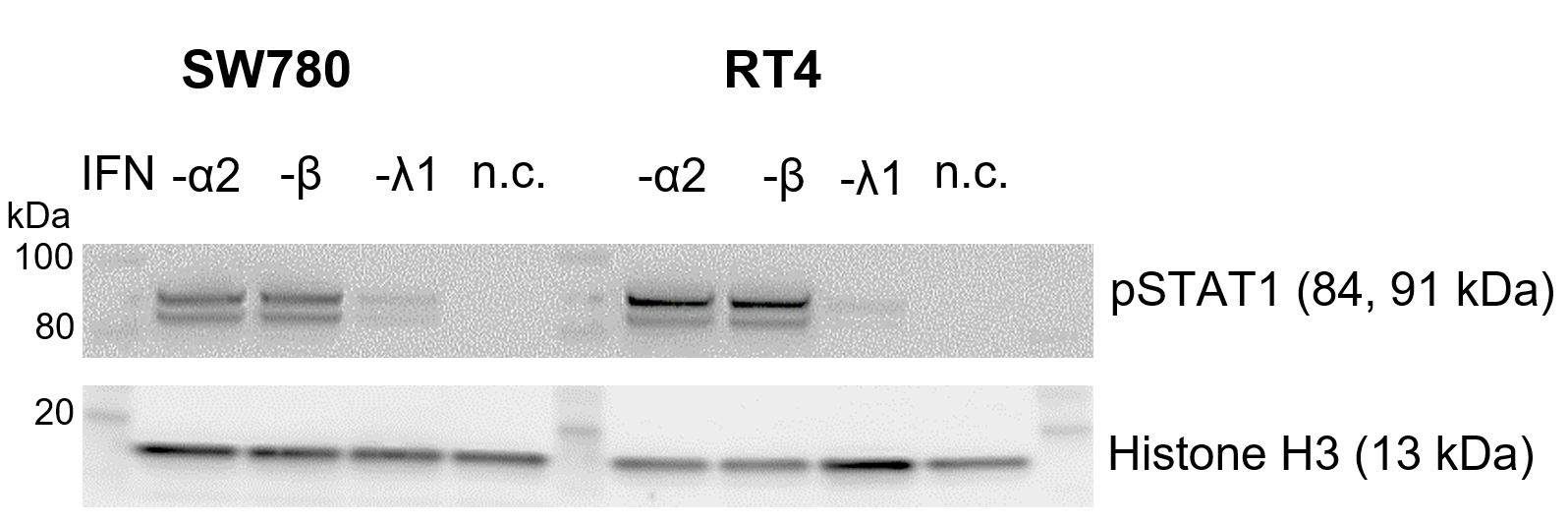 |
| **c - STAT2 - 30 min** | **d - pSTAT2 - 30 min** |
| 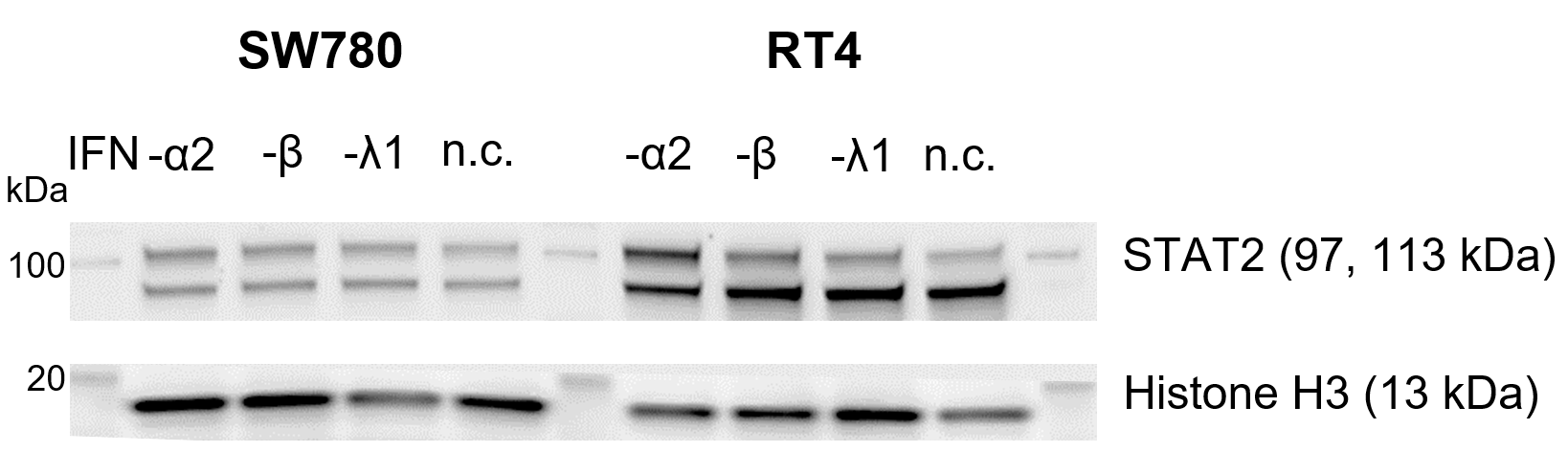 | 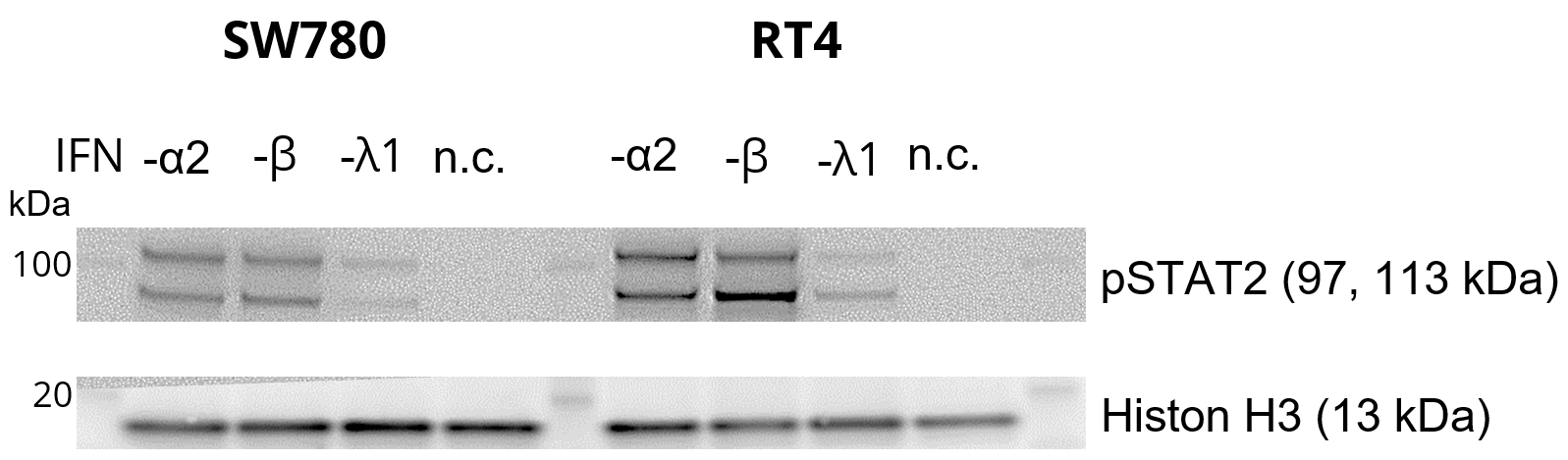 |

**Supplementary Material S5: Example** **western blot analysis of STAT1, STAT2, pSTAT1 and pSTAT2 in SW780 and RT4 cells after 30 min.** SW780 and RT4 cells were harvested after 30 min stimulation with 25,000 pg/ml IFN-α2, -β and -λ1, lysed and Western blot labeled with antibodies against (a) STAT1, (b) pSTAT1, (c) STAT2, (d) pSTAT2 and (a, b, c, d) histone H3 and detected via HRP-coupled secondary antibodies.
